# SARS‐CoV‐2 infections in cancer outpatients—Most infected patients are asymptomatic carriers without impact on chemotherapy

**DOI:** 10.1002/cam4.3435

**Published:** 2020-10-06

**Authors:** Louisa Hempel, Armin Piehler, Michael W. Pfaffl, Jakob Molnar, Benedikt Kirchner, Sebastian Robert, Julia Veloso, Beate Gandorfer, Zeljka Trepotec, Stefanie Mederle, Sabine Keim, Valeria Milani, Florian Ebner, Katrin Schweneker, Bastian Fleischmann, Axel Kleespies, Josef Scheiber, Dirk Hempel, Dietmar Zehn

**Affiliations:** ^1^ Sigmund Freud University Vienna Vienna Austria; ^2^ MVZ Freising Laboratory Freising Germany; ^3^ Division of Animal Physiology and Immunology School of Life Sciences Weihenstephan Technical University of Munich Munich Germany; ^4^ Cancer Center Dachau Dachau Germany; ^5^ Fraunhofer Institute of Optronics System Technologies, and Image Exploitation IOSB Karlsruhe Germany; ^6^ Department of Obstetrics Helios Clinic Pasing München Germany; ^7^ Oncological Center Fürstenfeldbruck Dachau Germany; ^8^ Oncological Center Donauwoerth Donauwoerth Germany; ^9^ BioVariance GmbH Waldsassen Germany; ^10^ Steinbeishochschule Berlin Berlin Germany

## Abstract

Oncologic patients are regarded as the population most at risk of developing a severe course of COVID‐19 due to the fact that malignant diseases and chemotherapy often weaken the immune system. In the face of the ongoing SARS‐CoV‐2 pandemic, how particular patients deal with this infection remains an important question. In the period between the 15 and 26 April 2020, a total of 1227 patients were tested in one of seven oncologic outpatient clinics for SARS‐CoV‐2, regardless of symptoms, employing RT‐qPCR. Of 1227 patients, 78 (6.4%) were tested positive of SARS‐CoV‐2. Only one of the patients who tested positive developed a severe form of COVID‐19 with pneumonia (CURB‐65 score of 2), and two patients showed mild symptoms. Fourteen of 75 asymptomatic but positively tested patients received chemotherapy or chemo‐immunotherapy according to their regular therapy algorithm (±4 weeks of SARS‐CoV‐2 test), and 48 of 78 (61.5%) positive‐tested patients received glucocorticoids as co‐medication. None of the asymptomatic infected patients showed unexpected complications due to the SARS‐CoV‐2 infection during the cancer treatment. These data clearly contrast the view that patients with an oncologic disease are particularly vulnerable to SARS‐CoV‐2 and suggest that compromising therapies could be continued or started despite the ongoing pandemic. Moreover the relatively low appearance of symptoms due to COVID‐19 among patients on chemotherapy and other immunosuppressive co‐medication like glucocorticoids indicate that suppressing the response capacity of the immune system reduces disease severity.

## Graphical abstract

Largely unexpected, only 78 of 1227 tested cancer patients showed a COVID‐19 infection of which only three patients were symptomatic, ranging from mild symptoms to pneumonia. These data clearly contrast the view that oncology patients are particularly vulnerable to SARS‐CoV‐2 and suggest that necessary chemotherapies could be continued or started despite the ongoing pandemic. One explanation could be the immunosuppressive effect of simultaneous systemic therapy which will be further investigated.

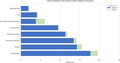

## 1 BACKGROUND

The clinical course of SARS‐CoV‐2 varies widely and ranges from asymptomatic infection to acute lung failure and death.^1^, ^2^ So far, the physiological conditions for this high variability in the clinical course are still largely unknown.^2^ A major challenge in the ongoing SARS‐CoV‐2 pandemic is to determine how co‐morbidities and therapies, that affect the response capacity of the immune system, impact the severity and treatment course of COVID‐19. Oncology is a field of particular relevance. The tumor itself and especially the treatments against it, such as chemotherapy or immunotherapy, significantly alter the ability of the immune system to respond to SARS‐CoV‐2. Cancer patients are suspected to be highly vulnerable to SARS‐CoV‐2 infections.^3^ As part of the containment of the pandemic, extensive social distancing and hygiene measures have been implemented in oncology outpatient clinics and hospitals.^4^ This led to a significant reduction in the number of outpatient and inpatient tumor therapies. Moreover to counter and minimize the risk of SARS‐CoV‐2 infection and severe complications, adjuvant chemotherapies, surgeries and other compromising therapies were eventually postponed or changed.^5^ It is still unclear whether oncological patients (with or without active disease and with or without current therapy) harbor a higher risk of SARS‐CoV‐2 infection, and potentially of developing more severe forms of COVID‐19. Furthermore, there is currently only scarce knowledge in the literature whether an infection affects essential treatment, or whether therapy increases the risk of COVID‐19, respectively. Recently, Liang et al. observed that COVID‐19 positive patients, with a history of cancer, had a higher risk of adverse complications such as being admitted to the intensive care unit, which requires invasive ventilation, or ultimately death compared to patients without cancer history (39% vs. 8%).^6^ However, there are still no generally accepted guidelines cancer patients treating physicians, such as surgeons and oncologists, can refer to when choosing anti‐cancer treatments during the SARS‐CoV‐2 pandemic. In the current study, most oncologic patients infected with SARS‐CoV‐2 were asymptomatic and showed no adverse effects to anti‐cancer therapy that could be related to COVID‐19.

## 2 MATERIAL AND METHODS

Between 15 and 26 April 2020, we conducted SARS‐CoV‐2 testing of oncological patient by RT‐PCR. All patients visiting one of the seven participating oncological outpatient clinics were tested for SARS‐CoV‐2, regardless of symptoms, resulting in a total of 1227 patients screened for SARS‐CoV‐2 infection.

In all clinics, infection protection measures according to the local health authorities were strictly followed. The patients treated in the outpatient clinics were constantly advised to act accordingly to basic hygiene rules and social distancing as recommended by the governmental authorities. SARS‐CoV‐2 carriers were consequently shielded from other patients, but nevertheless received individual treatment. Radiological (e.g. X‐ray or CT) or nuclear medicine (e.g. PET/CT or SPECT/CT) scans of the chest were not taken of each positively tested patient, but only in patients with symptoms suggesting a clinically significant affection of the lungs.

All SARS‐CoV‐2 negative patients received their therapy as planned before the virus outbreak. Thus, an ongoing therapy for all tumor patients could be assured. COVID‐19‐related symptoms such as cough, shortness of breath, loss of taste and temperature above 37.5°C were registered for each patient visiting the outpatient clinic.

Throat swabs were taken using CWY076 containing virus‐inactivating reagents from CWBIO (Amsterdam, Netherlands). To increase staff safety, samples were heat‐inactivated for 30 minutes at 60°C. Afterwards, RNA was isolated using the MGIEasy Magnetic Beads Virus DNA/RNA Extraction Kit in combination with an extraction protocol on MGI SP‐960 machines in a 96 well format. Extracted RNA was analyzed by RT‐qPCR using the BGI Real‐time fluorescent RT‐PCR kit for detecting 2019‐nCoV2 on Applied Bioscience ABI7500 instruments according to the vendor's manual. The target region in the procedure is located within in SARS‐CoV‐2 ORF1ab, and human GAPDH served as an internal reference for effective RNA isolation and RT‐PCR. Positive and negative controls were included in each run.

Differences of clinical characteristics of the patients between the two subgroups (positive and negative SARS‐CoV‐2‐PCR results) were tested for statistical significance using the Chi‐square test. Due to multiple testing, test results were adjusted using the Bonferroni method. Adjusted *P*‐values < .05 were regarded as statistically significant.

## 3 RESULTS

In the period between 15 and 26 April 2020, a total of 1227 patients were tested for SARS‐CoV‐2 by RT‐PCR. The demographic and clinical description of the entire population and of the positively and negatively tested sub‐populations, respectively, is shown in Table 1.

**TABLE 1:** Clinical features of CoV‐2 tested patients

	All patients (*n* = 1227)	Patients who tested positive (*n* = 78)	Patients who tested negative (*n* = 1149)	*P* value	Adjusted *P* value
Age in year^a^	62 (±16)	61 (±19)	62 (±17)	0.6910	0.6910
Sex				0.0891	0.0891
Male	522 (43%)	26 (33%)	496 (43%)		
Female	705 (57%)	52 (67%)	653 (57%)		
Mortality				0.9035	0.9035
Survived	1213 (99%)	77 (99%)	1136 (99%)		
Died	14 (1%)	1 (1%)	13 (1%)		
Disease type					
Solid cancer	755 (62%)	48 (62%)	707 (62%)	0.9991	1.0000
M0	242 (20%)	23 (30%)	219 (19%)	0.0251	0.1004
M1	177 (14%)	25 (32%)	152 (13%)	0.0000	0.0000
MX	336 (27%)	0 (0%)	336 (29%)	0.0000	0.0000
Cancer type					
Lip, oral cavity and pharynx	13 (1%)	1 (1%)	12 (1%)	0.8427	1.0000
Digestive organs	203 (17%)	14 (18%)	189 (16%)	0.7302	1.0000
Respiratory and intrathoracic organs	68 (6%)	4 (5%)	64 (6%)	0.8689	1.0000
Bone and articular cartilage	5 (0.4%)	0 (0%)	5 (0.4%)	0.5594	1.0000
Melanoma (skin)	18 (2%)	2 (3%)	16 (1%)	0.4049	1.0000
Mesothelial and soft tissue	26 (2%)	2 (3%)	24 (2%)	0.7779	1.0000
Breast	239 (20%)	12 (15%)	227 (20%)	0.3455	1.0000
Female genital organs	55 (5%)	6 (8%)	49 (4%)	0.1568	1.0000
Male genital organs	47 (4%)	7 (9%)	40 (4%)	0.0144	0.2016
Urinary tract	35 (3%)	3 (4%)	32 (3%)	0.5859	1.0000
Eye, brain and other parts of central nervous system	9 (0.7%)	0 (0%)	9 (0.8%)	0.4327	1.0000
Thyroid and other endocrine glands	11 (0.9%)	3 (4%)	8 (0.7%)	0.0043	0.0602
Hematological/lymphatic malignancies	323 (26%)	18 (23%)	305 (27%)	0.5010	1.0000
Non malignant hematological diseases	149 (12%)	13 (17%)	136 (11%)	0.2063	1.0000
Unknown or unspecified site^b^	25 (2%)	0 (0%)	25 (2%)	−	−
No information	331 (27%)	0 (0%)	331 (29%)	−	−
Comorbidities					
Hypertension	323 (26%)	17 (22%)	306 (27%)	0.3479	1.0000
Nicotine abuse	112 (9%)	7 (9%)	105 (9%)	0.9612	1.0000
Diabetes	156 (13%)	10 (13%)	146 (13%)	0.9767	1.0000
Chronic obstructive pulmonary disease	61 (5%)	5 (6%)	56 (5%)	0.5458	1.0000
Peripheral artery disease	25 (2%)	2 (3%)	23 (2%)	0.7337	1.0000
Rheumatoid arthritis	8 (0.7%)	1 (1%)	7 (0.6%)	0.4749	1.0000
Heart failure	44 (4%)	2 (3%)	42 (4%)	0.6160	1.0000
Cerebral infarction, stroke	42 (3%)	4 (5%)	38 (3%)	0.3920	1.0000
Myocardial infarction	31 (3%)	3 (4%)	28 (2%)	0.4428	1.0000
Hypercholesterolemia	49 (4%)	4 (5%)	45 (4%)	0.5969	1.0000
No information	715 (58%)	49 (63%)	637 (55%)	−	−
Comedication					
Glucocorticoid	714 (58%)	48 (62%)	666 (58%)	0.5356	1.0000
Bisoprolol	178 (15%)	8 (10%)	170 (15%)	0.2707	1.0000
Ramipril	158 (13%)	13 (17%)	145 (13%)	0.3018	1.0000
Zometa	157 (13%)	8 (10%)	149 (13%)	0.4879	1.0000
ASS	141 (12%)	7 (9%)	134 (12%)	0.4713	1.0000
HCT	136 (11%)	9 (12%)	127 (11%)	0.8949	1.0000
Others	628 (51%)	40 (51%)	588 (51%)	0.9854	1.0000
No information	240 (20%)	15 (19%)	225 (20%)	–	–
Cancer treatment^c^					
Chemotherapy	240 (20%)	23 (30%)	217 (19%)	0.0224	0.1792
Chemoimmunotherapy	173 (14%)	8 (10%)	165 (14%)	0.3135	1.0000
Antihormone therapy	145 (12%)	6 (8%)	139 (12%)	0.2435	1.0000
Immunotherapy	119 (10%)	3 (4%)	116 (10%)	0.0711	0.5688
TKI	52 (4%)	2 (3%)	50 (4%)	0.4483	1.0000
Bisphosphonate	26 (2%)	2 (3%)	24 (2%)	0.7779	1.0000
Surgery	190 (16%)	15 (19%)	175 (15%)	0.3447	1.0000
No systemic oncological therapy	75 (6%)	28 (36%)	47 (4%)	0.0000	0.0000
No information	433 (35%)	0 (0%)	433 (38%)	−	−

a Mean (± SD).

b ICD‐10‐Codes C76.‐ and C80.

Treatments in the past 6 mo.

**FIGURE 1:**
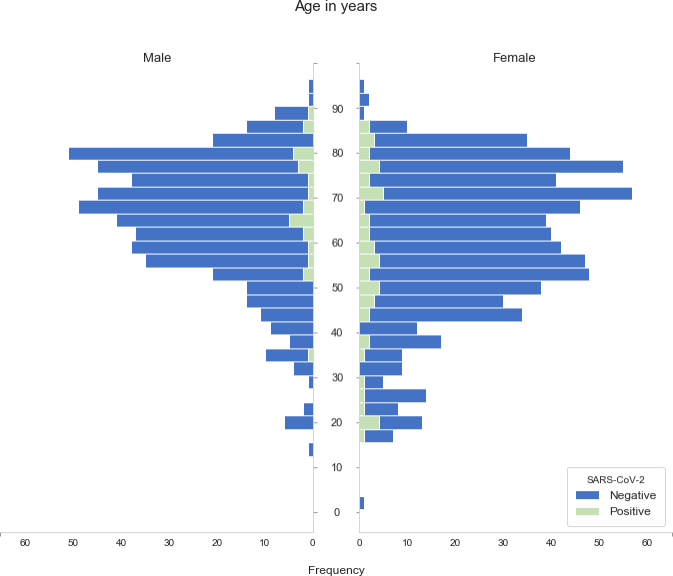
Gender distribution among SARS‐CoV2 tested patients

Seventy‐eight (6.4%) patients were tested positive. Of all negative tested patients, 56.8% (653/1149) were female and 43.2% (496/1149) male, compared to 66.7% (52/ 78) female and 33.3% (26/78) male in patients with positive CoV‐2 test, respectively (Figure 1). The average age of the patients in the positively tested group was 61 years. In the negative group, the average age was 62 years. The distribution of age and sex is shown in Table 1.

Only two (2.6%) of the 78 positively tested patients had mild symptoms such as shortness of breath and common cold symptoms at the time of testing, but these symptomatic patients did not suffer from any active tumor disease, neither received systemic therapy. Both patients were therapy‐naive and afebrile. One additional, positive tested patient (1.3%) was diagnosed with pneumonia requiring hospitalization, but did not require assisted ventilation according to a low CURB‐65 index. This patient suffered from an active tumor disease and received chemotherapy at the time of testing. Due to the patient's medical condition, the chemotherapy had to be aborted. Recently, the patient's COVID‐19 related symptoms have resolved, and chemotherapy has been continued. All symptomatic patients showed an ECOG performance status of 0 except for the hospitalized patient who showed an ECOG performance status of 1.

**FIGURE 2:**
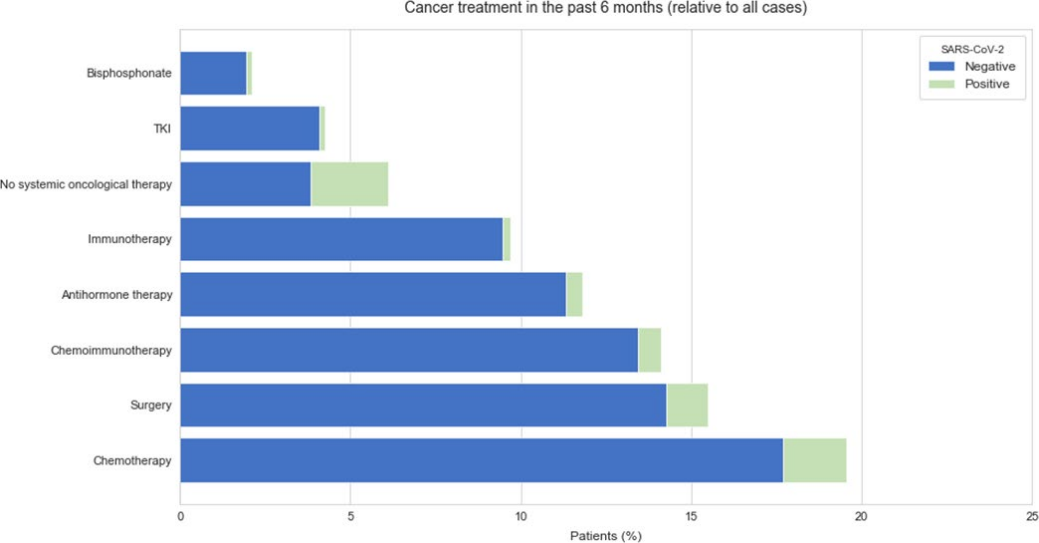
Cancer treatment of CoV‐2 tested patients within 6 mo before testing

The majority of patients with a positive test result (75/78) were solely asymptomatic virus‐carriers (96,2%). Forty‐four (56.4%) of the positively tested patients were undergoing systemic therapy: 23 (29.5%) chemotherapy, 6 (7.7%) antihormonal therapy, 8 (10.3%) chemotherapy in combination with immunomodulating antibodies (Rituximab/Obinutuzumab/Atezulizumab), 3 immunotherapy (3.8%), and two (2.6%) tyrosin kinase inhibitor (TKI) (Figure 2, Table 1). All listed treatments were applied within 6 months before testing.

**FIGURE 3:**
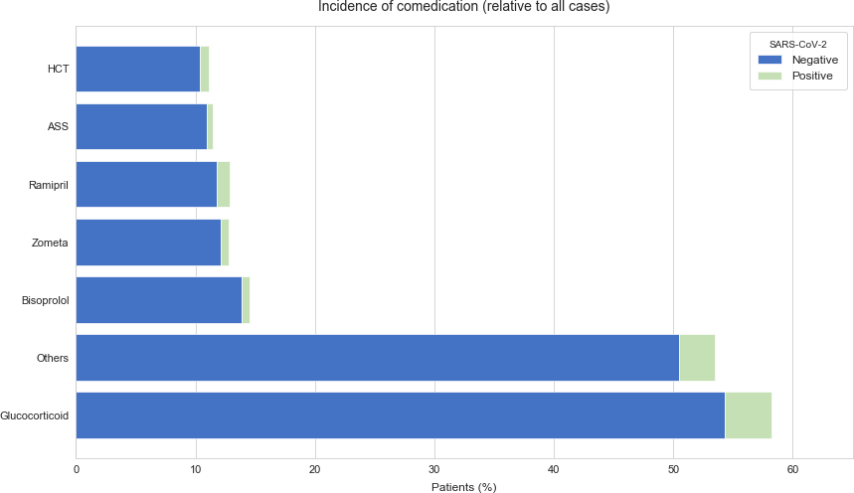
Co‐medication of CoV‐2 tested patients within 6 mo before testing

In the positively tested group, the majority (61.5%, 48/78) of the patients received glucocorticoids (Dexamethason or Prednisolon) as a co‐medication. 16.7% (13/78) of positively tested were under hypertension therapy with Ramipril, and thiazide diuretics were applied in 11.5% (9/78) (Figure 3, Table 1).

Both of the patients with mild symptoms were female and received no systemic anti‐cancer therapy. The patient who suffered from SARS‐CoV2‐related pneumonia was male, 77 years old and under treatment with a monoclonal antibody.

Distribution of cancer treatment and co‐medication of the negatively tested group is shown in Table 1 for comparison.

Among the different cancer types, the most present in patients with positive SARS CoV‐2 result were hematological and lymphatic malignancies (18/78, 23.1%), followed by non‐malignant hematological diseases (13/78, 16.7%). In the group of solid cancer, malignancies of digestive organs were reported most (14/78, 17.9%), followed by breast cancer (12/78, 15.4%) and malignancies of male genital organs (7/78, 9.0%). Twenty‐five of the positively tested patients who suffered from solid cancer disease, were reported with a stage M1 (32.1%) cancer disease and 23 with M0 (29.5%) (Table 1). For comparison of the cancer entity and stage distribution among the negatively tested patients, see Table 1.

The most common co‐morbidities among positively tested patients were hypertension (17/78, 21.8%), diabetes (10/78, 12.8%) and nicotine abuse (7/78, 9.0%) (Table 1).

Comparing the two subgroups, that is, all patients with positive and negative SARS‐CoV‐2 PCR test result, significant differences were only observed for the fraction of patients exhibiting metastasis (32% (25/78) M1 in patients with positive SARS‐CoV‐2 test result compared to 13% (152/1149) in the group with negative test result) and undergoing no systemic oncological therapy (36% (28/78) in the positively tested group compared to 4% (47/1149) in the negatively tested group).

## 4 DISCUSSION

Contrasting our expectation of a disadvantageous disease outcome in oncology patients, the evaluation of our data showed that the majority of the oncological patients who received a positive SARS‐CoV‐2 test result were asymptomatic carriers of SARS‐CoV‐2 despite ongoing anti‐cancer therapy. This is particularly noteworthy since it was previously assumed that these patients would have a particularly severe course or at least become symptomatic in the event of infection.^3^ Particularly surprising is the high frequency of asymptomatic carriers in our study, which is much higher than the rates detected among the systematic screenings performed, for instance on the French aircraft carrier Charles de Gaulle. Here, only 50% of positively tested persons remained free of symptoms. While these numbers need to be viewed with substantial precaution, it is particularly interesting that patients whose immune system was weakened by chemotherapy have such a low tendency to develop symptomatic diseases, which sounds counterintuitive at first glance. The observed lower disease incidence in oncology patients offers a completely new perspective on the possible underlying mechanisms. So far, no genetic associations with the development of severe COVID‐19 have been described. At the same time, there is a clear correlation among healthy individuals between disease severity and patient age.^7^, ^8^, ^9^ In fact, children rarely develop symptomatic COVID‐19 while the severity increases with age in healthy individuals.^9^, ^10^ The reason could be an association between the experience level of the immune system and disease severity. Aged individuals have a more diverse history of infections, which may include significant exposure to other corona viruses and cross‐reactivity between SARS‐CoV‐2 and other viruses. A complex infection history does not always make individuals more resistant to infection. Instead, it can also enhance disease severity compared to young individuals with a less complex infection history and less complex immune memory. We are therefore taking into consideration, that chemotherapy in cancer patients might have erased parts of the immunological memory. Cancer patients might therefore develop a lower disease incidence or severity level. There are numerous examples including childhood diseases or puberty related infection, such as EBV, where the same pathogen causes severe and sometimes life‐threatening infection in adults compared to young individuals.^11^, ^12^, ^13^ Furthermore, a number of studies have characterized how previously acquired non‐protective immunity increases disease severity in a subsequent infection.^14^ This includes serial dengue virus infections, where infections with one of the four different serotypes all cause a mild infection. In contrast, a subsequent re‐infection with a different strain can cause life‐threatening hemorrhagic fever.^14^ Similarly, augmented disease severity was observed in a failed RSV vaccine trial, where non‐neutralizing antibodies even caused a lethal outcome in vaccinated children.^15^, ^16^ Furthermore, patients who received vaccination against HIV showed increased infection rates in the STEP study.^16^, ^17^ The observations in the oncology patients suggest that the same may happen in SARS‐CoV‐2. There are also rare cases where pre‐existing immunity to related pathogens does not increase but dampens the ability of the immune system to respond to a subsequent infection. This includes the 1918 influenza pandemic, where a particular prior infection history is thought to have caused the unusual high incidence of severe cases among middle aged, 15‐45 years old individuals.^18^, ^19^ If pre‐existing immunity to other corona viruses or virus cross‐reactivity play a role in the pathogenicity of COIVD‐19 remains to be determined. If it does, then it is also possible that such disease enhancing pre‐existing immunity might have been diminished through chemotherapy. It has to be noted that our data do not provide a basis for explanation of the degree of severity of COVID‐19 in different individuals, and that further and more in‐depth studies on the patho‐mechanisms of COVID‐19 are needed.

It is also noteworthy that most patients with an asymptomatic course of CoV2‐infection in our study received glucocorticoids (dexamethasone or prednisolone). This is in accordance with a recent press release from the RECOVERY‐study showing that patients receiving glucocorticoid therapy had an advantageous outcome reducing deaths by one‐third in ventilated patients.^20^ It requires thorough follow up observations to explore which specific disease stages and treatments regimes this decreased severity applies to includes the use of chemotherapy and of check‐point inhibitors as well as immunomodulatory drugs.

In our study, radiological (e.g. X‐ray or CT) or nuclear medicine (e.g. PET/CT or SPECT/CT) scans of the chest were not regularly taken of positively tested patient. However, as several publications showed that asymptomatic COVID‐19 patients may exhibit alterations in radiological (e.g. X‐ray or CT) or nuclear medicine (e.g. PET/CT or SPECT/CT) scans of the chest (Refs. [^20^, ^21^]), it would be interesting to include these kind of diagnostics in future studies investigating asymptomatic patients.^5^, ^21^ A study in Italy found an interesting outcome. Oncological patients who underwent regularly scheduled radiological/nuclear medical scans for evaluation of tumor progression in some cases showed signs of pneumonia. A following RT‐PCR test on the SARS‐CoV2 came out positive although the patients did not show symptoms at the time but partly developed dyspnea and breathing difficulty later on. This might indicate that chest scans can be helpful in evaluating the course of an infection even if patients remain asymptomatic at first.^22^, ^23^_._

When looking at our data, it must also be considered that the current study started screening four weeks after the start of the lockdown in Bavaria, Germany. Therefore, the presented data are limited to the situation of social distancing in Bavaria.

## 5 CONCLUSION

As the majority of cancer patients have a suppressed immune system due to therapy, it has been assumed so far, that this makes them particularly vulnerable to SARS‐CoV‐2 infection, and in particular developing symptomatic cases of COVID‐19. The real‐world data we collected in our cohort, consisting only of patients treated in oncological outpatient clinics, contrasts strongly with this hypothesis. On the contrary to previous assumptions, only a minority (3/78) of positively tested tumor patients showed symptoms. Based on this observation, it should be investigated in the further course of this pandemic whether tumor patients are even less susceptible to the symptomatic course of a SARS‐CoV‐2 infection. To substantiate this thesis, however, further data and validation are needed, especially in larger cohorts of tumor patients.

## CONFLICT OF INTEREST

Louisa Hempel, Jakob Molnar, Sebastian Robert, Julia Veloso, Zeljka Trepotec, Sofie English, Philip Weinzierl, Valeria Milani, Kathrin Schweneker, Bastian Fleischmann, Josef Scheiber, Axel Kleespies, Wolfgang Kaminski, Dirk Hempel and Armin Piehler have no conflict of interest. Human Rights statements and informed consent: All procedures were in accordance with the ethical standard of the responsible committee of the Bavarian Chamber Of Physician (BLÄK) with the ethic committee´s approval No. 20037 and with the Helsinki Declatation of 1964 and it´s later amendments.

## AUTHOR CONTRIBUTIONS

Louisa Hempel, Dietmar Zehn, Jakob Molnar and Dirk Hempel conceived and designed the study. Sebastian Robert, Julia Veloso, Josef Scheiber and Louisa Hempel acquired the data. All authors discussed the results and contributed the final manuscript. Louisa Hempel carried out the experiment with the help from Armin Piehler, Zeljka Trepotec, and Beate Gandorfer. Valeria Milani, Katrin Schweneker, Bastian Fleischmann, Axel Kleespies, Sabine Keim, Stefanie Mederle, and Florian Ebner collected the samples. Michael Pfaffl and Benedikt Kirchner helped supervise the project.

## Data Availability

The data that support the findings of this study are available on request from the corresponding author. The data are not publicly available due to privacy or ethical restrictions.
